# Nuclease dead Cas9 is a programmable roadblock for DNA replication

**DOI:** 10.1038/s41598-019-49837-z

**Published:** 2019-09-16

**Authors:** Kelsey S. Whinn, Gurleen Kaur, Jacob S. Lewis, Grant D. Schauer, Stefan H. Mueller, Slobodan Jergic, Hamish Maynard, Zhong Yan Gan, Matharishwan Naganbabu, Marcel P. Bruchez, Michael E. O’Donnell, Nicholas E. Dixon, Antoine M. van Oijen, Harshad Ghodke

**Affiliations:** 1 School of Chemistry and Molecular Bioscience and Molecular Horizons, University of Wollongong, Wollongong, New South Wales 2522 Australia; 2 Illawarra Health and Medical Research Institute, Wollongong, New South Wales 2522 Australia; 3 Howard Hughes Medical Institute, Rockefeller University, New York, NY 10065 USA; 4 Department of Chemistry and Molecular Biosensors and Imaging Center, Carnegie Mellon University, 4400 Fifth Avenue, Pittsburgh, Pennsylvania 15213 USA; 5 Department of Biological Sciences, Carnegie Mellon University, 4400 Fifth Avenue, Pittsburgh, Pennsylvania 15213 USA

**Keywords:** Enzyme mechanisms, Single-molecule biophysics, Replisome, Stalled forks

## Abstract

Limited experimental tools are available to study the consequences of collisions between DNA-bound molecular machines. Here, we repurpose a catalytically inactivated Cas9 (dCas9) construct as a generic, novel, targetable protein–DNA roadblock for studying mechanisms underlying enzymatic activities on DNA substrates *in vitro*. We illustrate the broad utility of this tool by demonstrating replication fork arrest by the specifically bound dCas9–guideRNA complex to arrest viral, bacterial and eukaryotic replication forks *in vitro*.

## Introduction

Enzymes that regulate and execute the reactions that govern life must contend with a host of other DNA binding proteins as they perform their functions. Obtaining a detailed mechanistic understanding of how these reactions are performed in conditions approaching physiological contexts demands an exquisite ability to precisely manipulate strand and substrate occupancy by DNA binding proteins. Several examples of roadblocks are described in the literature that have proven invaluable for interrogating a variety of molecular mechanisms – from understanding how site-specifically bound proteins may confine the diffusion of proteins translocating on DNA, to blocking the enzymatic activity of transcription elongation complexes, or determining whether enzymes such as ring-shaped helicases can transiently open to overcome barriers on DNA^1–5^.

The impediment of the progress of DNA replication machinery on template DNA occupied by proteins is an important case in point. DNA replication occurs on chromosomal DNA while processes such as DNA repair, recombination and transcription continue. Replisomes encounter three major types of protein barriers: transcription complexes, nucleoid-associated proteins, and recombination filaments^6–8^. Successful replication across such roadblocks requires the coordinated action of several accessory factors and DNA-repair and dedicated restart proteins. Improper resolution of arrested forks can lead to replication fork collapse and eventually, genetic instability^3,9,10^.

Several roadblocks have been developed to mimic encounters between replication forks and protein barriers. Inspired by the Tus-*ter* block that terminates replication in *Escherichia coli*, replication fork arrest has been studied at *ter* sites recombined into the *Saccharomyces cerevisiae* chromosome^11^. Other approaches have involved the introduction of repeat sequences that enable binding of transcription factors to artificially introduce repressor/operator arrays, or proteins that polymerize to form nucleoprotein filaments^4,12–14^. Despite their tremendous utility in studying replication fork arrest, these methods suffer from several disadvantages: since the tandem binding of several roadblock proteins is required for effective stalling of the replication fork, the exact positions of the block are often poorly defined. Further, tedious recombination procedures are required to incorporate tandem arrays of terminator or repressor/operator sequences. Finally, high local concentrations of the fluorescently tagged roadblock may influence the local structure of the DNA due to a residual ability for the genetic fluorescent protein fusion to oligomerize. These limitations call for the development of a generic fluorescent protein roadblock that is monomeric, binds DNA with high affinity and specificity, and does not require extensive genetic manipulation of template DNA. Here, we describe the construction and validation of a fluorescently tagged nuclease dead Cas9 construct that serves as a monomeric roadblock for use in *in vitro* assays. Nuclease dead Cas9 blocks the progress of replication forks from viral, bacterial and eukaryotic model replisomes reconstituted *in vitro*.

## Results

### Construction of a stable roadblock that can be observed on long timescales *in vitro* and *in vivo*

**Figure 1:**
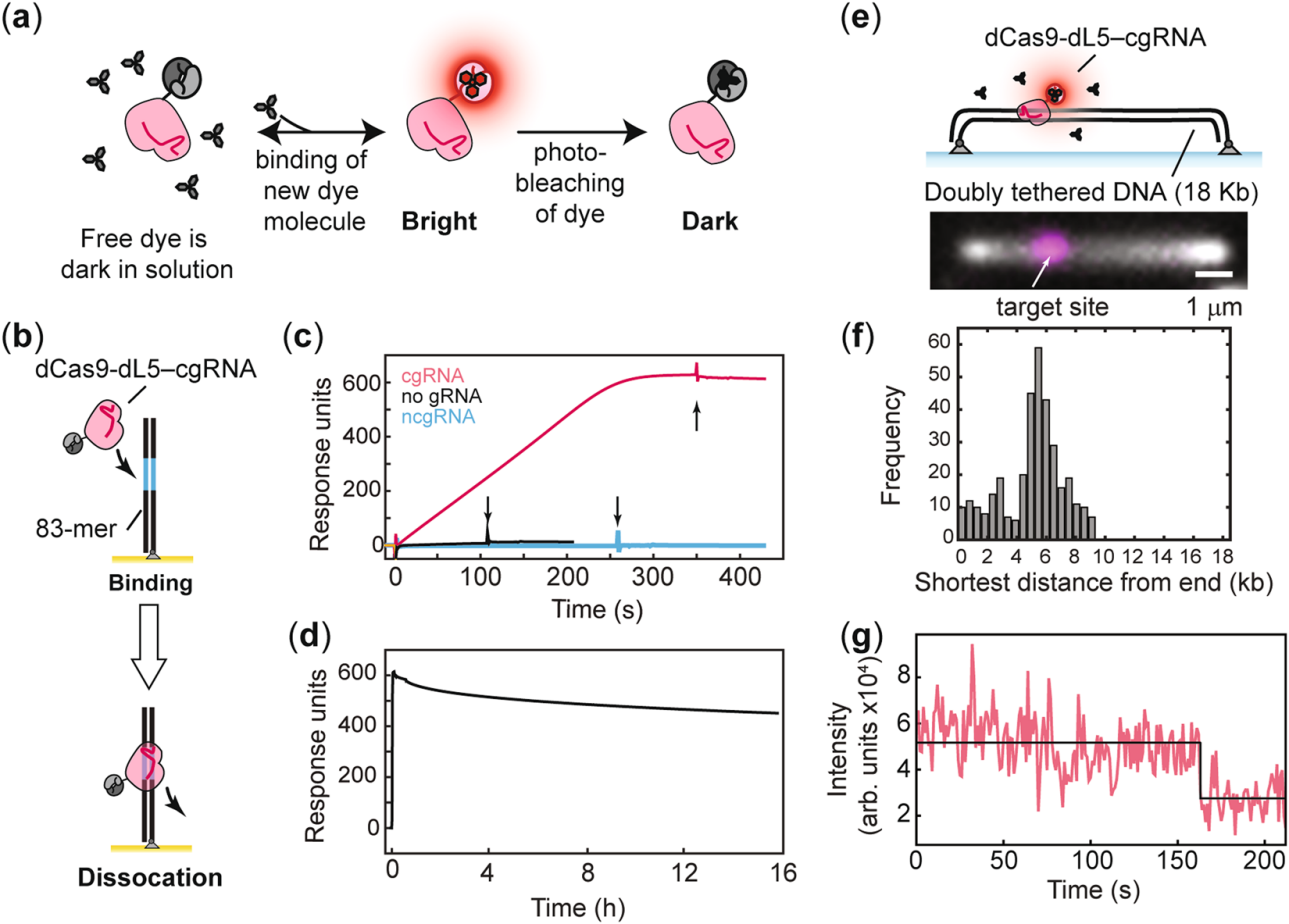
Characterization of dCas9-dL5. (**a**) Schematic of the dCas9-dL5 probe. Free dye is dark in solution. Binding of MGE to the dL5 tag enables visualization of dCas9-dL5. (**b**) Schematic of dCas9-dL5 binding to immobilized dsDNA containing the target sequence on an SPR chip. (**c**) Sensorgram describing the binding of dCas9-dL5 to dsDNA substrate carrying the target sequence in the absence of gRNA or programmed with a complementary gRNA (cgRNA1) or non-complementary gRNA (ncgRNA). Arrows indicate the completion of the injection phase, and switch to running buffer. *N* = 1 independent experiment. (**d**) Dissociation of dCas9-dL5–cgRNA1 bound to the dsDNA target monitored over 16 h. (**e**) Schematic and examples of elongated surface bound and elongated linear dsDNA template bound to dCas9-dL5 (scale bar – 1 μm). dsDNA is stained using Sytox orange, and dCas9-dL5–cgRNA1 is stained by MGE. (**f**) Histogram of detected position of dCas9-dL5–cgRNA1 complex visualized by addition of MGE (*n* = 345 molecules). The shortest distance to the position of the dCas9-dL5 is plotted here. (**g**) Example photo-bleaching trajectory of dCas9-dL5–cgRNA1–MGE complex (*n* = 345).

We reasoned that target bound, catalytically inactivated *Streptococcus pyogenes* Cas9 (dCas9) could act as a versatile roadblock enabling easy and precise targeting, and control over site-, orientation- and strand-specific binding to template DNA. Additionally, to permit long-term visualization of nucleic acid processing enzymes at sites of dCas9 roadblocks *in vitro*, we genetically fused dCas9 to the photostable fluoromodule dL5 that becomes fluorescent upon binding the dye, malachite green^15,16^. The fluorogen used in this work is an ester modified variant of the malachite green dye, herein referred to as malachite green-ester (MGE) (Fig. 1a).

### *In vitro* characterization of dCas9-dL5 binding to DNA

First, we purified the dCas9-dL5 fusion protein (Supplementary Table S1 and Fig. S1) and assayed its binding to an 83-mer target DNA using surface plasmon resonance (SPR) (Fig. 1b, Supplementary Table S2). Biotinylated target DNA was immobilized on a streptavidin-coated surface and a solution containing dCas9-dL5 pre-programmed with a complementary guide RNA (cgRNA1) was introduced (Fig. 1b, Supplementary Table S3). The dCas9-dL5–cgRNA1 complex exhibited robust and stable binding to the target DNA, whereas dCas9-dL5 alone or in presence of a non-complementary gRNA (ncgRNA) did not exhibit appreciable binding (Fig. 1c, Supplementary Table S3). We found that highly purified dCas9-dL5 alone exhibited binding to 83-mer biotinylated dsDNA in the absence of guide RNA (Fig. 1c), consistent with previous work^17^. This minimal binding was lost when dCas9-dL5 was programmed with ncgRNA and may reflect non-specific association of dCas9-dL5 for dsDNA ends. Importantly, dCas9-dL5–cgRNA associated strongly and stably with the target DNA – only approximately 25% of the bound complexes dissociated over 16 h (Fig. 1d; Supplementary Methods).

Next, we confirmed that dCas9-dL5 binds specifically to its target sequence. We used single-molecule total internal reflectance fluorescence (TIRF) microscopy to directly visualize dCas9-dL5 bound to its target sequence on individual DNA molecules. DNA molecules were pre-incubated with dCas9-dL5–cgRNA and doubly tethered to a streptavidin-coated glass coverslip inside a microfluidic flow cell using biotinylated oligonucleotide handles (Fig. 1e)^18^. Addition of MGE into the flow cell enabled visualization of the dL5 tag, and positioning of the dCas9-dL5–cgRNA complex along the length of the DNA (Supplementary Methods; 349 out of 899 DNA templates had dCas9-dL5 bound). Consistent with previous work, the position of the bound dCas9-dL5–cgRNA complex was in good agreement with the expected position (Fig. 1f)^17^. The spread in the position of the dCas9 may be attributable to incomplete extension of the doubly-tethered substrates on the surface of the flow cell. The use of the MGE allowed us to reliably visualize target-bound dCas9-dL5 for several minutes (Fig. 1g).

### Target-bound dCas9-dL5 blocks DNA replication in bulk measurements

**Figure 2:**
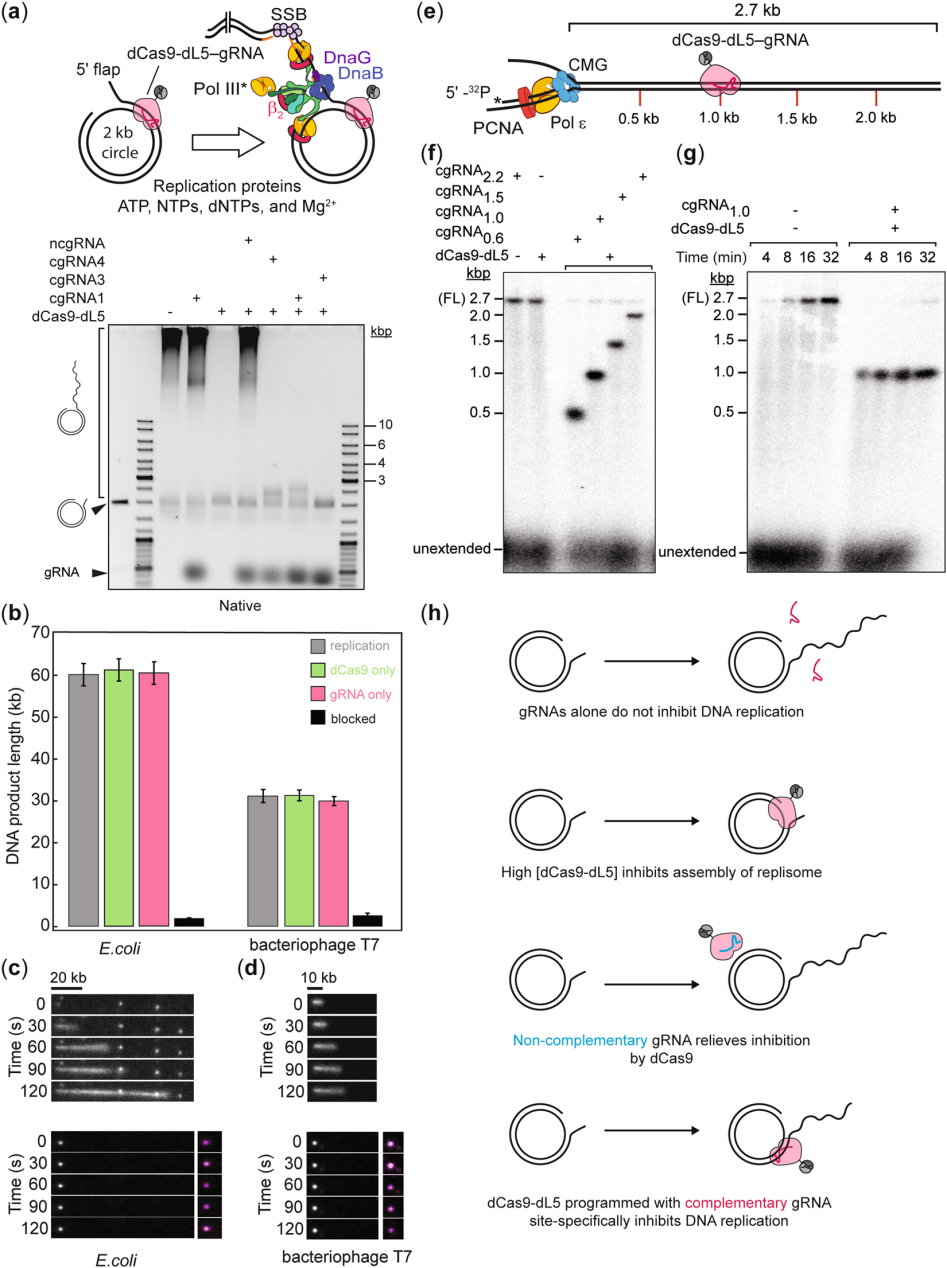
dCas9-dL5 efficiently and stably blocks bacterial, viral, and eukaryotic DNA replication regardless of the targeted strand. (**a**) Schematic of the leading-lagging rolling-circle DNA replication assay. Addition of the *E. coli* or T7 replication proteins, nucleotides, and Mg^2+^ initiates DNA synthesis in a leading strand replication reaction. The DNA products are separated by gel electrophoresis and visualized by staining with SYBR-Gold, or visualized by single-molecule fluorescence microscopy by staining with Sytox orange. dCas9-dL5 (100 nM) programmed with ncgRNA (400 nM) and cgRNAs alone (400 nM) alone do not inhibit DNA replication. At high concentrations, dCas9-dL5 (100 nM) alone inhibits DNA synthesis. dCas9-dL5 programmed with cgRNAs arrest the progress of the replication fork at the target site. See also, Fig. S2 (**b**) Bar plots of mean DNA product lengths from *E. coli* and T7 single-molecule rolling-circle DNA replication assays. Values plotted are derived from exponential fits to single-molecule DNA product length distributions (*n* > 91 molecules). Error bars indicate errors of the fit. (**c**,**d**) (Top panel) Example kymographs of an individual DNA molecule undergoing DNA replication by *E. coli* (c) (*n* = 177 molecules; replication efficiency of 26 ± 2% (SEM)) and T7 replisomes (d) (*n* = 136 molecules; replication efficiency of 24 ± 2% (SEM)) in the absence of target bound dCas9-dL5. (Bottom panels) Example kymographs of an individual DNA molecule arrested by target bound dCas9-dL5. The grey scale indicates the fluorescence intensity of stained DNA and magenta indicates dCas9-dL5–cgRNA stained by MGE. No replication events were detected. (**e**) Schematic of the eukaryotic DNA replication assay. Eukaryotic replication is blocked by dCas9-dL5 at specific positions on the replication template. (**f**) dCas9-dL5 efficiently blocks eukaryotic replication. The cgRNAs used to specifically target the template are indicated. cgRNA_0.6_ and cgRNA_2.2_ block the leading strand; cgRNA_1.0_ and cgRNA_1.5_ block the lagging strand (see Supplementary Methods for details). All reactions were stopped at 16 min. (**g**) Time course of eukaryotic replication in the presence or absence of dCas9-dL5 and cgRNA_1.0_. Reactions were stopped at 4, 8, 16 and 32 min as indicated. (**h**) Summary of interactions of dCas9-dL5 and template DNA. Only the correctly programmed dCas9-dL5-cgRNA complex site specifically inhibits DNA replication.

These observations highlight the potential of dCas9-dL5 to be applied as a general roadblock to study details of molecular transactions on DNA *in vitro*. As a proof of principle, we ran reconstituted replisomes from model systems into this dCas9 roadblock. First, we investigated whether single dCas9-dL5-cgRNA (either cgRNA1, cgRNA3 or cgRNA4) molecules bound to template DNA could impede DNA replication using a rolling-circle replication assay, both at the ensemble and single-molecule levels^19–23^. This assay allows observation of robust DNA synthesis by replisomes under a variety of experimental conditions (Fig. 2a, see Supplementary Fig. S3 for raw data). Pre-incubation of template DNA with dCas9-dL5-cgRNA1 resulted in potent replication fork arrest of reconstituted *E. coli* replisomes during either leading-strand (Fig. 2a and Supplementary Fig. S2) or simultaneous leading- and lagging-strand DNA synthesis (Supplementary Fig. S2), with an average blocking efficiency of 85 ± 2% (*N* [replicates] = 5). Importantly, neither complementary gRNA alone (Fig. 2a and Supplementary Fig. S2) nor dCas9-dL5 alone (Fig. 2a, see Supplementary Fig. S2 and Supplementary Methods) or programmed with ncgRNA (Fig. 2a and see Supplementary Fig. S2) could site-specifically arrest DNA replication (summarized in Fig. 2h). Further, dCas9-dL5 targeted to the leading strand using a complementary gRNA duplex (cgRNA4 (Ld)) blocked *E. coli* leading-strand (Fig. 2a) and leading- and lagging-strand synthesis with similar efficiencies (85 ± 2% (*N* [replicates] = 5) (see Supplementary Fig. S2). Taken together, these observations demonstrate that encounters of the replisome with either the PAM-proximal (cgRNA1 (Lg)) or PAM-distal (cgRNA4 (Ld)) side of bound dCas9-dL5–cgRNA complexes does not influence its ability to arrest replication. Notably, dCas9-dL5-cgRNA3 that targets the replisome assembly site inhibited leading-strand DNA replication (Fig. 2a).

### Target-bound dCas9-dL5 blocks DNA replication in single-molecule assays

Next, to demonstrate the use of this tool in single-molecule assays, we repeated these experiments in single-molecule rolling-circle assays and measured the average lengths of DNA products synthesized by individual *E. coli* replisomes in the presence of dCas9-dL5–cgRNA complexes. Consistent with the bulk experiments, target bound dCas9-dL5 was found to specifically block simultaneous leading- and lagging-strand DNA synthesis (Fig. 2b,c).

Finally, we examined the capacity of dCas9-dL5 as a universal roadblock for arresting replication forks site-specifically; the ability of dCas9-dL5 programmed with complementary gRNA to arrest replication *in vitro* was assessed using model replisomes from T7 bacteriophage (Fig. 2b,d) and *S. cerevisiae* (Fig. 2e–g, see Supplementary Fig. S3 for raw data and Supplementary Methods online). Replication reactions using both reconstituted replisomes carried out in the presence of template associated dCas9-dL5–cgRNA also exhibited replication fork arrest as observed with *E. coli*. Taken together, these results demonstrate that the dCas9-dL5–cgRNA complex binds with high specificity and stability to its target DNA sequence and can be visualized effectively in a variety of experimental conditions.

## Discussion

Here, we have harnessed the specificity and programmability of the CRISPR/Cas9 system and combined it with the photo-stability of the dL5 fluoromodule to repurpose dCas9 as a tool for studying metabolic processes that occur on DNA. Our *in vitro* characterization of dCas9-dL5 binding to dsDNA indicated that this tool is a high-stability and sequence-specific roadblock. Indeed, the stability of this roadblock could be affected by PAM-distal mismatches between the gRNA and DNA, as shown previously in investigations of wild-type Cas9 stability^24,25^. Interestingly, replication fork arrest is not strand-specific and occurs when dCas9-dL5 is targeted to either the leading- or lagging-strands, suggesting that the dCas9-gRNA complex may inhibit strand separation by the replicative helicase. This is in contrast to recent investigations showing that the elongating RNA polymerase is able to displace wild-type or nuclease dead Cas9 proteins stably bound to the template strand, but not on the non-template strand^26,27^. Here, we demonstrate the suitability of the dCas9-dL5 tool for investigating mechanisms that underlie the protein dynamics that govern replication fork rescue at sites of protein roadblocks on template DNA undergoing replication by viral, bacterial, and eukaryotic replisomes. Indeed, this precisely tunable roadblock may prove useful in understanding fine mechanistic details of DNA helicase and translocases, repair and other sliding factors involved in DNA metabolism.

## Materials and Methods

### Replication proteins

*E. coli* DNA replication proteins were produced as described previously: the β_2_ sliding clamp^28^, SSB^29^, the DnaB_6_(DnaC)_6_ helicase–loader complex^30^, DnaG primase^31^, the Pol III τ_3_δδ’χψ clamp loader^32^ and Pol III αεθ core^22^. *S. cerevisiae* DNA replication proteins were produced as described previously: the CMG (Cdc45/Mcm2-7/GINS) helicase^33^, the Mrc1–Tof1–Csm3 (MTC) complex^34^, DNA polymerase Pol ε^33^, the PCNA sliding clamp^35^, RPA^33^ and the RFC clamp loader^36^. T7 gp2.5 was produced as described previously^37^. Highly purified T7 gp4 helicase and DNA polymerase gp5/trx were generous gifts of Charles Richardson.

### DNA and RNA oligonucleotides

DNA oligonucleotides and tracrRNA, unmodified crRNAs and crRNAs containing Alexa Fluor 555 were purchased from Integrated DNA Technologies (USA). Sequences of DNA oligonucleotides, crRNAs and tracrRNAs used in this study are listed in Table S2. Synthetic guide RNA (gRNA) targeting various regions of the 2.7 kb linear DNA template were produced with the EnGen sgRNA Synthesis Kit (New England Biolabs, USA) using the DNA are also described in the Table S2. All DNA and RNA oligonucleotides were stored in TE buffer (10 mM Tris-HCl pH 8.0, 1 mM EDTA) at –20 °C.

### Construction of plasmid pJL001

Plasmid pJL001 was constructed by ligation of a 1007 bp *Sac*I–*Xho*I gene block (Aldervon, USA) between the corresponding sites in pET302 (obtained from Addgene plasmid #72269), to encode dCas9-dL5 containing an N-terminal 6xHis and C-terminal 3xFLAG tags. The corresponding plasmid sequence is given in Table S1.

### Expression and purification of dCas9-dL5

*E. coli* strain Rosetta 2(DE3) containing plasmid pJL001 was grown in LB medium supplemented with thymine (25 mg/L) and ampicillin (100 μg/mL) at 37 °C. Upon growth to *A*_600_ = 0.8, the temperature was reduced to 16 °C and protein expression induced by addition of 0.5 mM isopropyl-β-D-thiogalactoside. Cultures were further shaken for 16 h at 16 °C, then chilled on ice. Cells (8 g from 2 L of culture) were harvested by centrifugation, frozen in liquid nitrogen and stored at –80 °C. All subsequent steps were carried out in a cold room maintained at 6 °C. After thawing, cells were resuspended in lysis buffer (20 mM Tris-HCl pH 7.6, 0.1 mM EDTA, 1 mM dithiothreitol, 150 mM NaCl, 5% (*v/v*) glycerol) and 2x Protease Inhibitor Cocktail tablets and 0.7 mM phenylmethylsulfonyl fluoride were added to inhibit proteolysis. Cells were lysed by being passed twice through a French press (12,000 psi), and cell debris were then removed by centrifugation. Crude supernatant (85 mL) was brought to 0.4% (*v/v*) in polyethylenimine (PEI) and vigorously stirred. After 40 min, the white precipitate was separated by centrifugation. The remaining pellet was homogenized by stirring in lysis buffer for 15 min. The remaining white precipitate was immediately collected by centrifugation and the supernatant discarded. The remaining pellet was further homogenized in lysis buffer +250 mM NaCl for 15 min. After centrifugation, the high salt supernatant containing dCas9-dL5 was collected yielding Fraction I (72 mL). Proteins that were precipitated from Fraction I by addition of solid ammonium sulfate (0.32 g/mL) and stirring for 60 min, were collected by centrifugation and dissolved in 30 mL of FLAG buffer (25 mM Tris-HCl pH 7.6, 1 mM EDTA, 1 mM dithiothreitol, 200 mM NaCl and 5% (*v/v*) glycerol). The solution was dialysed against 2 L of the same buffer overnight, to yield Fraction II. Fraction II was added to 4 mL FLAG M2 resin prepared as per manufacturer’s instructions and left to incubate with constant mixing. After 1 h, the FLAG M2 resin was poured into a PD-10 column and equilibrated in FLAG wash buffer (50 mM Tris-HCl pH 7.6, 0.5 mM dithiothreitol, 0.5 mM EDTA, 300 mM NaCl, 5% (*v/v*) glycerol). The column was washed with FLAG buffer until the *A*_280_ was approximately 0.05, and dCas9-dL5 was eluted using FLAG wash buffer containing 3X FLAG peptide (200 μg/mL). Fractions containing dCas9-dL5 were collected and pooled to yield Fraction III (15 mL), which was dialysed against 2 L of HisTrap buffer (50 mM Tris-HCl pH 7.6, 0.5 mM EDTA, 2 mM dithiothreitol, 300 mM NaCl, 20 mM imidazole pH 8.0, 5% (*v/v*) glycerol). Fraction III was applied at 1 mL/min onto a 5 mL HisTrap column equilibrated in HisTrap buffer. The column was washed until *A*_280_ returned to baseline and dCas9-dL5 was eluted as a single peak with a step elution of 300 mM imidazole pH 8.0. Fractions under the peak were pooled and dialysed against 2 L of storage buffer (50 mM Tris-HCl pH 7.6,1 mM EDTA, 3 mM dithiothreitol, 300 mM NaCl, 50% (*v/v*) glycerol) to give Fraction IV (4 mL, containing 6.9 mg of protein; Fig. S1A). Aliquots were stored at –20 °C.

### Rolling-circle replication template

DNA rolling circle substrates were prepared as previously described^38^.

### Linear DNA substrates

Plasmid pSupercos1 DNA^39^ (7 pmol) was linearized overnight at 37 °C with 100 U of *Bst*XI in 1 x buffer 3.1 (New England Biolabs, USA). The 18,345 bp fragment was purified with a Wizard SV gel and PCR clean up kit (Promega, USA) and the concentration was measured. DNA oligonucleotides (750 pmol of arm 1, 4500 pmol arm 2, and 70 pmol capping 1, 2) were annealed by heating at 94 °C for 5 min before slow cooling. The biotinylated handles were ligated to the 18,345 bp fragment in 1 X T4 ligase buffer and 2000 U of T4 ligase overnight at 16 °C. Biotinylated linear DNA substrates were purified from excess DNA oligonucleotides by adjusting NaCl to 300 mM and loaded by gravity onto a Sepharose 4B (1 × 25 cm) column, equilibrated in gel filtration buffer (10 mM Tris-HCl pH 8.0, 1 mM EDTA, and 300 mM NaCl). Biotinylated linear DNA substrates eluted as a single peak in the column void volume, fractions under the peak were analyzed by agarose gel electrophoresis. Fractions containing linear DNA substrates were pooled and dialysed overnight in 2 L of sterilized TE buffer, concentrated 2-fold in a vacuum concentrator and the concentration measured. This protocol typically yielded ~20 μg DNA. Aliquots were stored at –80 °C.

### Forked linear DNA substrates

The eukaryotic DNA replication template, a linearized 2.7 kb plasmid ligated to a synthetic replication fork, was prepared as previously described^33,40^. A synthetic 37-mer oligonucleotide (Fork primer) was end-labeled with ^32^P-ATP by T4 polynucleotide kinase (New England Biolabs, USA) according to manufacturer’s instructions and annealed to the forked substrate by heating to 85 °C and slowly cooling.

### Assessment of dCas9 interactions by SPR

SPR measurements were carried out on a BIAcore T200 instrument (GE Healthcare, Sweden) at 20 °C in SPR buffer (30 mM Tris-HCl pH 7.6, 0.5 mM dithiothreitol, 0.25 mM EDTA, 0.005% (*v/v*) surfactant P20) containing NaCl/MgCl_2_ concentrations as described. A streptavidin-coated (SA) sensor chip was activated with three sequential injections of 1 M NaCl, 50 mM NaOH (40 s each at 5 µL/min). Then, a solution (2.5 nM) of the 3’-biotinylated 83-mer template dsDNA in SPR buffer containing 50 mM NaCl (SPR running buffer), assembled *in situ* by premixing 83-S and 83-AS oligonucleotides (to final concentrations of 1.2 and 1 μM, respectively) in hybridization buffer (20 mM Tris-HCl pH 7.8, 50 mM NaCl, 5 mM MgCl_2_) at 90 °C for 5 min followed by slow cooling overnight to the room temperature, was used to immobilize ∼150 RU of DNA template at 5 μL/min over 456 s onto the surface of flow cell 4, whereas flow cell 3 was left unmodified and served as a control (4–3 subtraction).

To interrogate binding specificity of dCas9-dL5 for immobilized 83 dsDNA template in the presence of complementary guide cgRNA1, a solution of protein (10 nM) with cgRNA1 (50 nM) in SPR buffer supplemented with 100 mM NaCl and 10 mM MgCl_2_ (SPR binding buffer) was made to flow at 10 μL/min for 350 s, yielding a response of ~625 RU (Fig. 1C). Following the association phase, the slow dissociation of protein from immobilized DNA template initiated by re-introduction of the running buffer in the flow cell and monitored over >70 s indicated stable binding. Bound proteins/RNA complexes were removed and immobilized dsDNA on the chip surface regenerated by three successive 40 s injections of 3 M MgCl_2_ at 10 µL/min. Injections of dCas9-dL5 under similar experimental conditions, either in the presence of ncgRNA (257 s injection) or in the absence of any guide gRNA (107 s), as well as the injection of cgRNA1 alone (66 s) led to barely detectable binding responses, suggesting that only the dCas9-dL5–cgRNA1 complex interacts stably and specifically with 83 template dsDNA. Furthermore, binding of dCas9-dL5–cgRNA1 is concentration dependent, since comparative injection of 30 nM dCas9-dL5 with 50 nM cgRNA led to faster association (Fig. S1A). Moreover, notably similar responses measured at equilibrium when 10 nM and 30 nM dCas9-dL5 were injected with 50 nM cgRNA1 (~625 RU) implies saturation of all the template DNA molecules on the chip surface with 10 nM dCas9-dL5–cgRNA1, indicating: (a) that the *K*_D_ for the dCas9-dL5–cgRNA1−dsDNA interaction is significantly below 10 nM in buffer containing 100 mM NaCl and 10 mM MgCl_2_, and (b) that the dCas9-dL5–cgRNA1 complex binds 83-mer template DNA in 1:1 molar ratio, *i.e*. considering that the ratio of mol. wt. between dCas9-dL5–cgRNA1 complex (~218.1 kDa; 184.5 kDa for dCas9 and ~33.6 kDa for cgRNA1) and template dsDNA (51.7 kDa; 25.5 kDa for 83-S and 26.2 kDa for 83-AS) is 4.2, and considering that ~150 RU of DNA was immobilized on the surface, ~630 RU (4.2·150 RU) of bound dCas9-dL5–cgRNA1 could be expected at saturation in case of 1:1 interaction with template DNA.

To demonstrate the strong association and long-term stability of dCas9-dL5–cgRNA1 complex with the target DNA template, the dissociation of a complex assembled on the surface during injection of 30 nM dCas9-dL5 and 50 nM cgRNA1 (as described above) from immobilized DNA in SPR running buffer, interspersed with an early 1500 s injection of SPR binding buffer to assess the complex stability in the buffer used for the association, was monitored for over 16 h (58807 s; final response was ∼450 RU; Fig. 1d). The surface (immobilized template dsDNA) was then regenerated with one 40 s injection of 3 M MgCl_2_ at 10 µL/min. Assuming first-order dissociation and SPR responses that were measured following the injection of SPR binding buffer, at the start of measured dissociation *R*_0_ = 575 RU and at the end *R*_t_ = 450 RU over the period of t = 57000 s, the dissociation half-life of >44 hours (see also Supplementary Methods) was calculated using Equation 1:

$${t}_{1/2}=\frac{t\cdot \,\mathrm{ln}\,2}{\mathrm{ln}\,\frac{{R}_{0}}{{R}_{{\rm{t}}}}}$$

### Measurement of dCas9-dL5 binding specificity on long DNA substrates

Microfluidic flow cells were prepared as described in “Preparation of flow-cells for *in vitro* imaging”. To help prevent non-specific interactions of proteins and DNA with all surfaces, they were blocked with 2% Tween20 in blocking buffer (50 mM Tris-HCl pH 7.6, 50 mM KCl). Imaging parameters are described in “*In vitro* single-molecule fluorescence microscopy”.

First, 9 nM dCas9-dL5 was incubated with 15 nM cgRNA1 at 37 °C for 5 min in reaction buffer (25 mM Tris-HCl pH 7.6, 10 mM MgCl_2_, 150 mM potassium glutamate, 0.1 mM EDTA and 0.0025% (*v/v*) Tween20). The dCas9-dL5–cgRNA1 complex was further incubated with 125 pM biotinylated linear DNA substrates at 37 °C for 20 min in reaction buffer supplemented with 0.5 mg/mL heparin. To reduce heterogeneity in DNA lengths upon binding to the surface, 200 µM chloroquine was added immediately prior to injection of the sample into the flow cell. The solution was injected at a constant rate of 17 µL/min until an appropriate DNA density was achieved. Next, the flow cell was washed with 2 mL of reaction buffer, supplemented with 100 mM NaCl, 15 nM gRNA and 0.5 mg/mL heparin. dCas9-dL5–cgRNA1–DNA complexes were imaged in reaction buffer containing 150 nM Sytox orange and 150 nM MGE.

### *In vitro* ensemble *E. coli* replication assays

Standard leading-strand replication assays were set up in replication buffer (RB; 60 mM Tris-HCl pH 7.6, 24 mM Mg(OAc)_2_, 100 mM potassium glutamate, 1 mM EDTA and 0.005% (*v/v*) Tween20) and contained 2 nM rolling-circle replication template, specified concentrations of dCas9-dL5 and gRNA, 60 nM DnaBC, 30 nM τ_3_δδ’χψ, 90 nM Pol III αεθ core, 200 nM β_2_, 10 mM dithiothreitol, 1 mM ATP, and 125 µM dNTPs in a final volume of 12 µL. First, dCas9-dL5 was incubated with gRNA for 5 min, and further incubated with rolling-circle DNA templates for 5 min at room temperature. Components (except dCas9-dL5–gRNA–DNA) were mixed and treated at room temperature, then cooled in ice for 5 min prior to addition of dCas9-dL5–gRNA–DNA complexes. Reactions were initiated at 30 °C and quenched at specified time points by the addition of 200 mM EDTA and 2% (*w/v*) SDS. The quenched reactions were loaded into a 0.6% (*w*/*v*) agarose gel in 2x TAE. Products were separated by agarose gel electrophoresis, at 60 V for 150 min and stained in SYBR-Gold (Invitrogen) and imaged under UV light.

*E. coli* leading- and lagging-strand DNA replication reactions were carried out as previously described^22^ with the following minor modifications. Reactions were set up in RB, and contained 4 nM rolling-circle replication template, specified concentrations of dCas9-dL5 and gRNA, 60 nM DnaBC, 80 nM DnaG, 30 nM τ_3_δδ’χψ, 10 nM SSB, 90 nM Pol III αεθ core, 200 nM β_2_, 10 mM dithiothreitol, 1 mM ATP, 125 µM dNTPs, and 250 µM NTPs to a final volume of 12 µL, quenched after 30 min by addition of 1.5 μL 0.5 M EDTA and 3 μL DNA loading dye (6 mM EDTA, 300 mM NaOH, 0.25% (*v*/*v*) bromocresol green, 0.25% (*v*/*v*) xylene cyanol FF, 30% (*v*/*v*) glycerol). DNA products were separated on a 0.6% (*w*/*v*) alkaline agarose gel at 14 V for 14 h. The gel was then neutralized in TAE buffer, stained with SYBR-Gold and imaged under UV light.

### *In vitro* ensemble T7 replication assays

T7 leading-strand DNA replication assays were carried out using previously described conditions^41^. Briefly, reactions were set up in T7 replication (TR) buffer (25 mM Tris-HCl pH 7.6, 10 mM MgCl_2_, 50 mM potassium glutamate, 0.1 mM EDTA and 0.0025% (*v/v*) Tween20) and contained 2 nM rolling-circle replication template, specified concentrations of dCas9-dL5 and cgRNA1, 180 nM gp2.5, 5 nM gp4, 40 nM gp5, 10 mM dithiothreitol, 1 mM ATP, 1 mM CTP, and 600 µM dNTPs, in a final volume of 12 µL. First, dCas9-dL5 was incubated with cgRNA1 for 5 min, and further incubated with rolling-circle DNA templates for a further 5 min at room temperature. Components (except dCas9-dL5–cgRNA1–DNA) were mixed and treated at room temperature, then cooled in ice for 5 min prior to addition of dCas9-dL5–cgRNA1–DNA complexes. Reactions were initiated at 30 °C and quenched at specified time points by the addition of 200 mM EDTA and 2% (*w/v*) SDS. The quenched reactions were loaded onto the 0.6% (*w/v*) agarose gel, which was run under the same conditions as standard *E. coli* leading-strand replication assays.

### *In vitro* ensemble *S. cerevisiae* replication assays

Leading-strand replication assays were set up in eukaryotic replication (ER) buffer (25 mM Tris-OAc pH 7.5, 5% glycerol, 80 μg/mL BSA, 5 mM tris(2-carboxyethyl)phosphine, 10 mM Mg(OAc)_2_, 50 mM potassium glutamate, 0.1 mM EDTA), and contained 1.5 nM DNA substrate (see section on Forked Linear DNA substrates), 30 nM CMG, 30 nM MTC, 20 nM Pol ε, 10 nM RFC, 30 nM PCNA, 600 nM RPA, 5 mM ATP and 120 μM dNTPs, and where indicated 40 nM sgRNA and 20 nM dCas9-dL5 in a final volume of 20 μL. First, DNA was incubated with CMG and MTC for 2 min at 30 °C followed by an additional 2 min with dCas9-dL5 and cgRNAs. Components except ATP and RPA were added and further incubated for 5 min at 30 °C. Replication was initiated by addition of ATP and RPA. The reactions proceeded for the indicated amount of time at 30 °C and were quenched with an equal volume of 2x stop solution (40 mM EDTA and 2% (*w/v*) SDS). DNA products were separated on a 1.3% (*w/v*) alkaline agarose gel at 35 V for 16 h. Gels were backed with DE81 paper (GE Healthcare), dried by compression, exposed to a phosphorimager screen, and imaged with a Typhoon FLA 9500 PhosphorImager (GE Healthcare).

### *In vitro* single-molecule fluorescence microscopy

*In vitro* single-molecule microscopy was performed on an Eclipse Ti-E inverted microscope (Nikon, Japan) with a CFI Apo TIRF 100x oil-immersion TIRF objective (NA 1.49, Nikon, Japan), as previously described^6^. The temperature was maintained at 31 °C (unless otherwise stated) by an electronically heated flow-cell chamber coupled to an objective heating jacket (Okolab, USA). NIS-elements was used to operate the microscope and the focus was locked through Perfect Focus System (Nikon, Japan). Images were captured using a Evolve 512 Delta EMCCD camera (Photometics, USA) with an effective pixel size of 0.16 μm. DNA molecules stained with 150 nM Sytox orange were imaged with a CW 568-nm Sapphire LP laser (200 mW max. output), and ET600/50 emission filter (Chroma, USA) at 0.76 W/cm^2^. dCas9-dL5–MGE complexes were imaged with a CW 647-nm OBIS laser (100 mW max. output), and 655LP emission filter (Chroma, USA) at 57.7 W/cm^2^.

### Preparation of flow-cells for *in vitro* imaging

Replication reactions were carried out in microfluidic flow-cells constructed from a PDMS flow chamber placed on top of a PEG-biotin-functionalized microscope coverslip as previously described^19,22,32,41^. Once assembled, all surfaces of the flow-cell including connecting tubing were blocked against non-specific binding by introduction of 1 mL malic acid buffer (100 mM Na.maleate pH 7.5 and 250 mM NaCl) containing 1% (*w/v*) blocking reagent (Roche, Switzerland).

### Single-molecule rolling-circle blocking replication assays

The overall experimental scheme was to first form the dCas9-dL5–cgRNA1–DNA complex. Next, dCas9-dL5–cgRNA1–DNA complex was attached via the 5’-biotinylated flap-primed 2030-bp dsDNA circle bearing a 25-nt fork gap, to the surface via a biotin–streptavidin bond. Following a wash to remove unbound dCas9-dL5, replication was initiated by continuous flowing of reconstituted replisomes, ATP, dNTPs, and rNTPs and flow-stretching the DNA.

Specifically, 10 nM dCas9-dL5 was incubated with 200 nM cgRNA1 for ~5 min at 37 °C in single-molecule imaging (SM) buffer (25 mM Tris-HCl pH 7.6, 10 mM Mg(OAc)_2_, 50 mM potassium glutamate, 0.1 mM EDTA, 10 mM dithiothreitol and 0.0025% (*v/v*) Tween20). The dCas9-dL5–cgRNA1 complex was then incubated with 100 pM replication templates for a further 20 min at 37 °C. The dCas9-dL5–cgRNA1–DNA complexes were adsorbed to the surface in SM buffer + 150 nM Sytox orange at 10 μL/min until an appropriate surface density was achieved. The flow-cell was then washed with 200 μL of SM buffer containing 50 mM NaCl. Following this replication was initiated — *E. coli* leading- and lagging-strand DNA replication reactions were carried out under the continuous presence of all proteins as previously described^22^. T7 leading- and lagging-strand DNA replication assays were carried out under the continuous presence of all proteins using previously described conditions^19^. All *in vitro* single-molecule rolling-circle blocking experiments were performed at least three times. Errors bars reported reflect the standard error of the mean (SEM).

### Analysis of agarose gels of replication products

Agarose gel images were adjusted for brightness and contrast for clear visualisation using FIJI^42^. Blocked replication products were quantified in FIJI using in-house built plugins, by comparing the integrated intensity of bands between control and reaction lanes; the resulting percentages were then corrected for background and for the specified control.

### *In vitro* image analysis

Image analysis was performed in FIJI, using the Single Molecule Biophysics plugins (available at https://github.com/SingleMolecule/smb-plugins). Raw videos (.nd2 format) were converted into TIF files and flattened with the excitation beam profile as described previously^43^. For quantification of DNA product lengths, intensity projections were generated by summing 10 frames to reduce the contribution of transverse Brownian fluctuations of the DNA. Product length was determined by deconvolving the length of the rolling-circle substrate using the calibrated pixel size in bp (here, 1 pixel = 470 bp). Product length distributions were fit with a single-exponential decay (assuming a single rate-limiting step determining the end of an event). All distributions were made and fitted using MATLAB (Mathworks, USA).

## Supplementary information

Supplementary Information

## Data Availability

The data that support the findings of this study are available from the corresponding authors upon reasonable request. Unique biological materials used in this study are available from the corresponding authors. Home-built ImageJ plugins used in this study are freely available on the Github repository for Single-molecule/Image analysis tools (https://github.com/SingleMolecule).
