# Supplementary material for: Nuclease dead Cas9 is a programmable roadblock for DNA replication
Source: Sci Rep. 2019 Sep 16;9:13292. doi: 10.1038/s41598-019-49837-z (PMC6746809; doi:10.1038/s41598-019-49837-z)
Supplement: Supplementary file 1 — Supplementary Information [file 41598_2019_49837_MOESM1_ESM.pdf]

Nuclease dead Cas9 is a programmable roadblock for DNA replication

Authors

Kelsey S. Whinn,[1,2]† Gurleen Kaur,[1,2]† Jacob S. Lewis[1,2], Grant D. Schauer[3], Stefan H. Müller[1,2], Slobodan Jergic[1,2], Hamish Maynard[1], Zhong Yan Gan[1], Matharishwan Naganbabu[4,5], Marcel P. Bruchez[4,5], Michael E. O'Donnell[3], Nicholas E. Dixon[1,2], Antoine M. van Oijen[1,2]*, Harshad Ghodke[1,2*]

Affiliations

1 – School of Chemistry and Molecular Bioscience and Molecular Horizons, University of Wollongong, Wollongong, New South Wales 2522, Australia

2 – Illawarra Health and Medical Research Institute, Wollongong, New South Wales 2522, Australia

3 – Howard Hughes Medical Institute, Rockefeller University, New York, NY 10065, USA

4 – Department of Chemistry and Center for Nucleic Acids Science and Technology, Carnegie Mellon University, 4400 Fifth Avenue, Pittsburgh, Pennsylvania 15213, USA

5 - Department of Biological Sciences and Molecular Biosensor and Imaging Center, Carnegie Mellon University, 4400 Fifth Avenue, Pittsburgh, Pennsylvania 15213, USA

*Corresponding author: Antoine van Oijen (vanoijen@uow.edu.au)
Harshad Ghodke (harshad@uow.edu.au)

† These authors contributed equally to this work

Supplementary Information

Supplementary Materials

Supplementary Methods

Assessment of diffusion limited binding kinetics of dCas9-dL5 binding to target containing DNA in SPR studies

The sensorgrams obtained during the association of dCas9-dL5-cgRNA at different concentrations of dCas9-dL5 (Fig. S1B) exhibited a distinct biphasic profile. The linear response (RU)–time (s) relationship suggests that the association of the complex from solution could be a fast, diffusion limited process. To examine this possibility, solutions of dCas9-dL5 (10 nM) with cgRNA1 (50 nM) in SPR buffer supplemented with 150 mM NaCl and 10 mM $MgCl_2$ were injected over immobilized template DNA for 120 s at three different flow rates accessible to the BIAcore T200 instrument: 20, 30 and 80 μL/min (Fig. S1C). The observed increase in association rates with the increase in flow rates confirms that the interaction is indeed diffusion-limited, a situation that occurs when the diffusion of analyte from the bulk solution to the chip surface is slower than its binding to the ligand. Conversely, it suggests that the dissociation of analyte (dCas9-dL5–cgRNA) from the ligand (83-mer DNA) must be also be a diffusion limited process, *i.e.* upon dissociation from the ligand, the analyte may not diffuse into bulk solution, allowing it to re-bind. This would result in an apparently slower dissociation rate; therefore, the dissociation half-life ($t_{1/2}$) of dCas9-dL5-cgRNA1 from its target dsDNA is an over-estimate of the true dissociation half-life.

Measurement of position of bound dCas9-dL5-cgRNA on 18-kb template

Measurement of the position of the bound dCas9-dL5–cgRNA complex on the 18-kb template was performed as follows:

1. Line profiles were manually drawn over all individual DNA molecules. The length of the individual molecules was defined as the distance between the maximum and minimum of the first derivative of the intensity along the drawn lines. Using these measurements, a length distribution was plotted, and values below the 25% and above the 75% percentile were classified as outliers. The resulting distribution was fit to a Gaussian distribution with a mean of 39.5 ± 0.1 pixels. This mean length was then assumed to correspond to the total length of 18,345 bp of the DNA substrate. This conversion resulted in a calibration factor of 466 ± 1 bp/pixel.
2. Next, peaks were detected along the line profile in the MGE-channel. The position of the detected peaks, relative to the ends of the DNA-molecules was then calculated. The distances to both ends of the DNA-molecule were measured. The position in base-pairs was calculated using the calibration described above. The histogram shows the smaller of the two distances from the DNA ends for each molecule.

Description of cgRNAs used in studies of bacterial DNA replication

Exact positions of targeting gRNA on the rolling-circle DNA replication template are as follows; cgRNA1 targeted to nucleotides 1402-1421 of the lagging-strand, cgRNA3

targeted to nucleotides 177-196 of the leading-strand, and cgRNA4 targeted to nucleotides 1046-1065 of the leading-strand.

Description of cgRNAs used in studies of eukaryotic DNA replication

Exact positions of targeting gRNA on the eukaryotic linear DNA replication template are as follows: $cgRNA_{0.6}$ targeted nucleotides 583–602 of the leading strand, $cgRNA_{1.0}$ targeted nucleotides 1005–1024 of the lagging strand, $cgRNA_{1.5}$ targeted nucleotides 1493–1512 of the lagging strand, and $cgRNA_{2.2}$ targeted nucleotides 2196–2215 of the leading strand.

Supplementary Figures

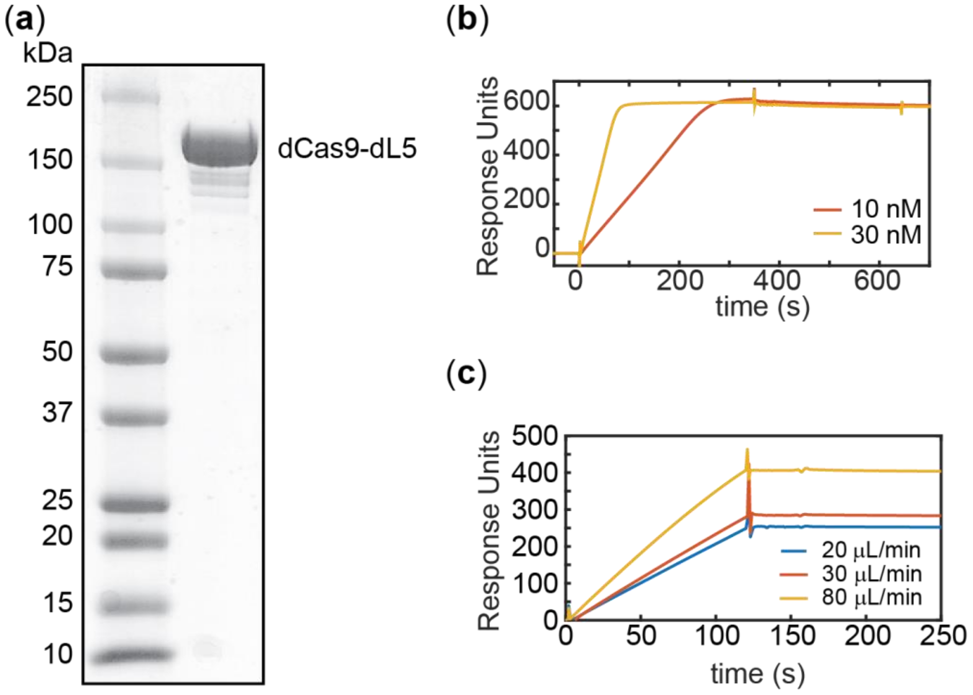

Figure S1: Characterization of dCas9-dL5. (**a**) Coomassie stained 4–20% SDS-PAGE of purified dCas9-dL5. (**b**) Sensorgrams showing binding to and dissociation of 10 and 30 nM dCas9-dL5-cgRNA from 83-mer dsDNA substrate immobilized on an SPR chip. (**c**) Sensorgrams monitoring the association of dCas9-dL5-cgRNA (10 nM) injected at three different flow rates (20, 30 and 80 μL/min) onto 83-mer dsDNA containing target sequence immobilized on an SPR chip. The linearity and difference in responses indicates mass transfer limitation. *N* = 1 independent experiment.

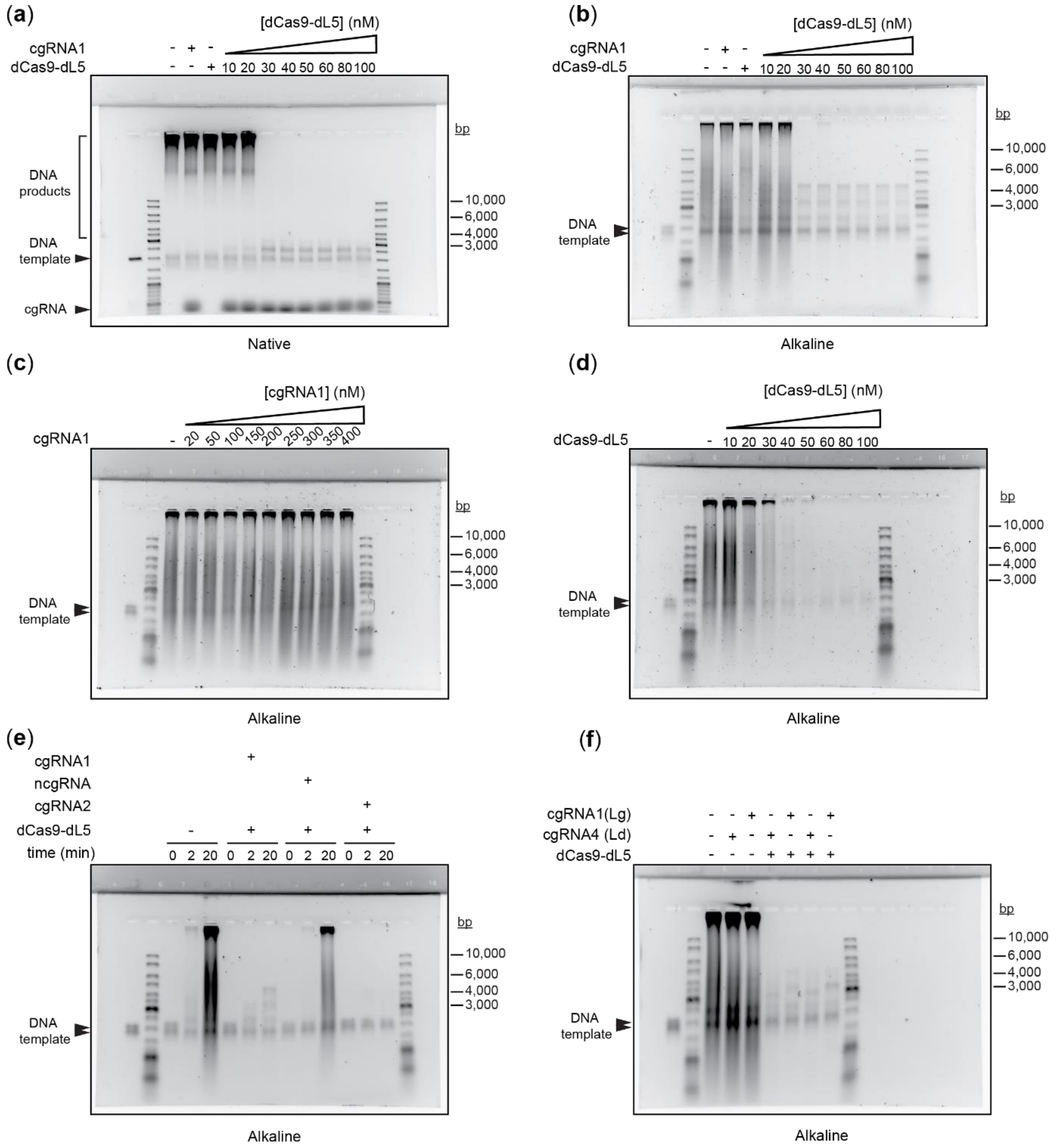

Figure S2: Target-bound dCas9-dL5 site-specifically arrests *E. coli* DNA synthesis. (**a**) Target-bound dCas9-dL5 arrests *E. coli* leading strand DNA synthesis. Unless otherwise specified reactions contained 400 nM cgRNA1 and 20 nM dCas9-dL5. At concentrations below 20 nM, dCas9-dL5 does not completely arrest leading-strand DNA synthesis. *N* > 3 independent experiments. (**b**) Target-bound dCas9-dL5 arrests *E. coli* leading- and lagging-strand DNA synthesis. Unless otherwise specified, reactions contained 400 nM cgRNA1. At concentrations below 20 nM, dCas9-dL5 does not completely arrest leading- and lagging-strand DNA synthesis. *N* > 3 independent experiments. (**c**) High concentrations of complementary gRNAs do not inhibit *E. coli* leading- and lagging-strand DNA synthesis. Unless otherwise specified, 50 nM dCas9-dL5 was used for all reactions. *N* > 2 independent experiments. (**d**) dCas9-dL5 alone does not site-specifically inhibit *E. coli* leading- and lagging-strand DNA synthesis. Non-specific inhibition is observed at high

concentrations of dCas9-dL5 alone (see Supplementary Methods, Fig. 2A and summary in Fig. 2H). *N* > 2 independent experiments. (**e**) Only dCas9-dL5 programmed with complementary gRNAs specifically arrests *E. coli* leading- and lagging-strand DNA synthesis. Reactions contained 50 nM dCas9-dL5 and 400 nM gRNAs. Reactions were initiated at 30°C and aliquots were removed and quenched at 0, 2, and 20 min time points. *N* > 3 independent experiments. (**f**) *E. coli* leading- and lagging-strand DNA synthesis arrest by target-bound dCas9-dL5 is not strand specific. Unless otherwise specified reactions contained 400 nM cgRNAs and 50 nM dCas9-dL5. Lg denotes cgRNA targeted to the lagging strand, and Ld denotes cgRNA targeted to the leading strand. *N* > 3 independent experiments. All panels show photographic negative images of gels that had been stained with SYBR-gold nucleic acid stain.

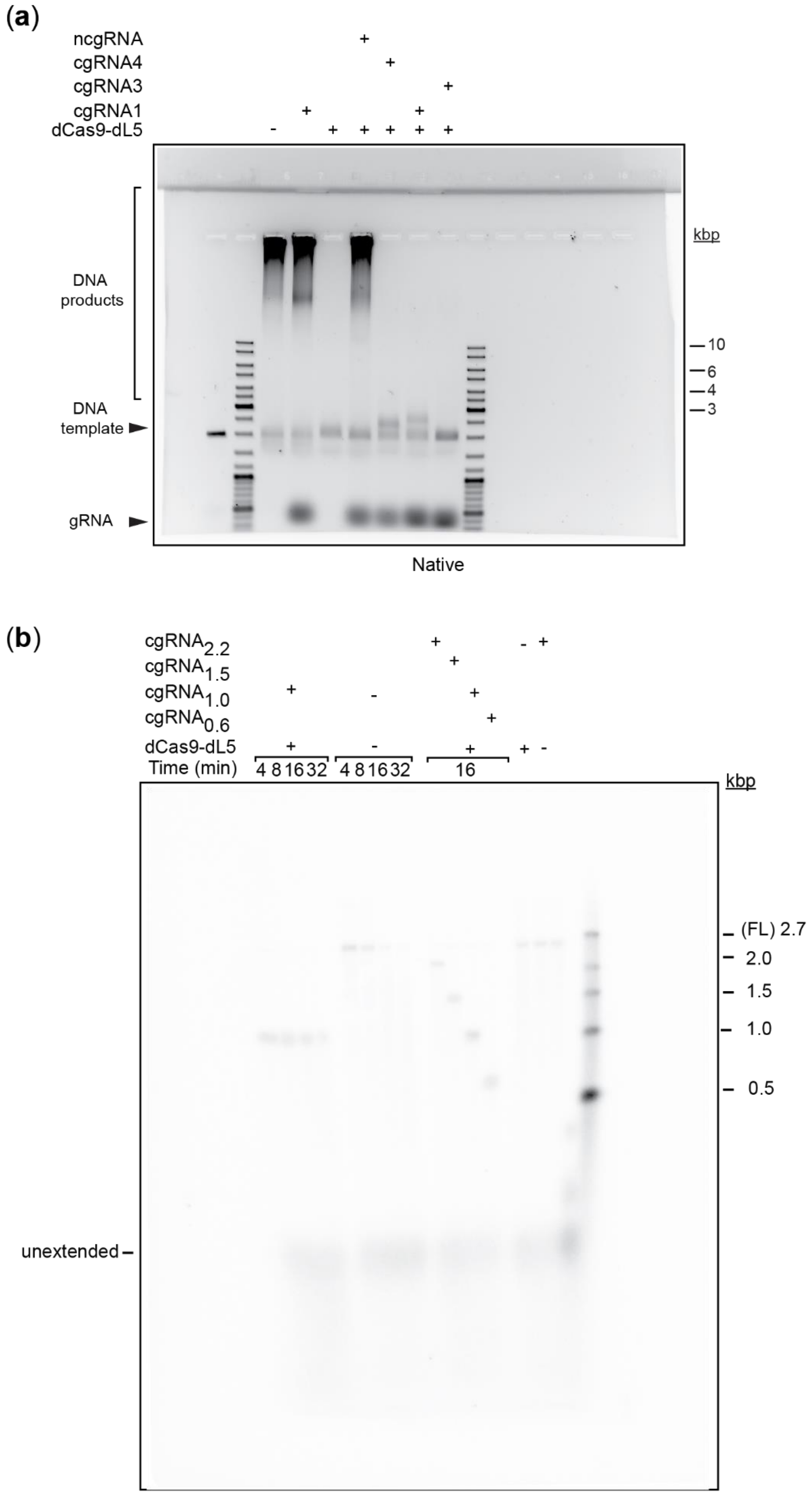

Figure S3: Raw data presented in Fig. 2. (a) dCas9-dL5 efficiently and stably blocks bacteria DNA replication regardless of the targeted strand. dCas9-dL5 (100 nM) programmed with cgRNAs (400 nM) arrest the progress of the bacterial replication fork at target sites. **(b)** dCas9-dL5 efficiently blocks eukaryotic replication at target sites

indicated by cgRNA, and time course assay of eukaryotic replication in the presence or absence of dCas9-dL5 and $cgRNA_{10}$.

Supplementary Tables

Supplementary Table 1: Sequence of plasmid used in this study

**pdCas9-dL5**
AGTAAAGCCCTCGCTAGATTTTAATGCGGATGTTGCGATTACTTCGCCA<br>ACTATTGCGATAACAAGAAAAAGCCAGCCTTTCATGATATATCTCCCAA<br>TTTGTGTAGGGCTTATTATGCACGCTTAAAAATAATAAAAGCAGACTTG<br>ACCTGATAGTTTGGCTGTGAGCAATTATGTGCTTAGTGCATCTAACGCT<br>TGAGTTAAGCCGCGCCGCGAAGCGGCGTCGGCTTGAACGAATTGTTA<br>GACATTATTTGCCGACTACCTTGGTGATCTCGCCTTTCACGTAGTGGA<br>CAAATTCTTCCAACTGATCTGCGCGCGAGGCCAAGCGATCTTCTTCTT<br>GTCCAAGATAAGCCTGTCTAGCTTCAAGTATGACGGGCTGATACTGGG<br>CCGGCAGGCGCTCCATTGCCCAGTCGGCAGCGACATCCTTCGGCGCG<br>ATTTTGCCGGTTACTGCGCTGTACCAAATGCGGGACAACGTAAGCACT<br>ACATTTCGCTCATCGCCAGCCCAGTCGGGCGGCGAGTTCCATAGCGT<br>TAAGGTTTCATTTAGCGCCTCAAATAGATCCTGTTCAGGAACCGGATCA<br>AAGAGTTCCTCCGCCGCTGGACCTACCAAGGCAACGCTATGTTCTCTT<br>GCTTTTGTCAGCAAGATAGCCAGATCAATGTCGATCGTGGCTGGCTCG<br>AAGATACCTGCAAGAATGTCATTGCGCTGCCATTCTCCAAATTGCAGTT<br>CGCGCTTAGCTGGATAACGCCACGGAATGATGTCGTCGTGCACAACA<br>ATGGTGACTTCTACAGCGCGGAGAATCTCGCTCTCTCCAGGGGAAGC<br>CGAAGTTTCCAAAAGGTCGTTGATCAAAGCTCGCCGCGTTGTTTCATC<br>AAGCCTTACGGTCACCGTAACCAGCAAATCAATATCACTGTGTGGCTT<br>CAGGCCGCCATCCACTGCGGAGCCGTACAAATGTACGGCCAGCAACG<br>TCGGTTCGAGATGGCGCTCGATGACGCCAACTACCTCTGATAGTTGAG<br>TCGATACTTCGGCGATCACCGCTTCCCTCATGATGTTTAACTTTGTTTT<br>AGGGCGACTGCCCTGCTGCGTAACATCGTTGCTGCTCCATAACATCAA<br>ACATCGACCCACGGCGTAACGCGCTTGCTGCTTGGATGCCCGAGGCA<br>TAGACTGTACCCCAAAAAAACAGTCATAACAAGCCATGAAAACCGCCA<br>CTGCGCCGTTACCACCGCTGCGTTCGGTCAAGGTTCTGGACCAGTTG<br>CGTGAGCGCATACGCTACTTGCATTACAGCTTACGAACCGAACAGGCT<br>TATGTCCACTGGGTTCGTGCCTTCATCCGTTTCCACGGTGTGCGTCAC<br>CCGGCAACCTTGGGCAGCAGCGAAGTCGAGGCATTTCTGTCCTGGCT<br>GGCGAACGAGCGCAAGGTTTCGGTCTCCACGCATCGTCAGGCATTGG<br>CGGCCTTGCTGTTCTTCTACGGCAAGGTGCTGTGCACGGATCTGCCCT<br>GGCTTCAGGAGATCGGAAGACCTCGGCCGTCGCGGCGCTTGCCGGT<br>GGTGCTGACCCCGGATGAAGTGGTTCGCATCCTCGGTTTTCTGGAAG<br>GCGAGCATCGTTTGTTCGCCCAGCTTCTGTATGGAACGGGCATGCGG<br>ATCAGTGAGGGTTTGCAACTGCGGGTCAAGGATCTGGATTTCGATCAC<br>GGCACGATCATCGTGCGGGAGGGCAAGGGCTCCAAGGATCGGGCCT<br>TGATGTTACCCGAGAGCTTGGCACCCAGCCTGCGCGAGCAGGGGAAT<br>TAATTCCCACGGGTTTTGCTGCCCGCAAACGGGCTGTTCTGGTGTTGC<br>TAGTTTGTTATCAGAATCGCAGATCCGGCTTCAGGTTTGCCGGCTGAA<br>AGCGCTATTTCTTCCAGAATTGCCATGATTTTTTCCCCACGGGAGGCG<br>TCACTGGCTCCCGTGTTGTCGGCAGCTTTGATTCGATAAGCAGCATCG<br>CCTGTTTCAGGCTGTCTATGTGTGACTGTTGAGCTGTAACAAGTTGTCT<br>CAGGTGTTCAATTTCATGTTCTAGTTGCTTTGTTTTACTGGTTTCACCTG<br>TTCTATTAGGTGTTACATGCTGTTCATCTGTTACATTGTCGATCTGTTCA<br>TGGTGAACAGCTTTGAATGCACCAAAAACTCGTAAAGCTCTGATGTAT

CTATCTTTTTTACACCGTTTTCATCTGTGCATATATGGACAGTTTTCCCT
TTGATATGTAACGGTGAACAGTTGTTCTACTTTTGTTTGTTAGTCTTGAT
GCTTCACTGATAGATACAAGAGCCATAAGAACCTCAGATCCTTCCGTAT
TTAGCCAGTATGTTCTCTAGTGTGGTTCGTTGTTTTTGCGTGAGCCATG
AGAACGAACCATTGAGATCATACTTACTTTGCATGTCACTCAAAAATTT
TGCCTCAAAACTGGTGAGCTGAATTTTTGCAGTTAAAGCATCGTGTAGT
GTTTTTCTTAGTCCGTTATGTAGGTAGGAATCTGATGTAATGGTTGTTG
GTATTTTGTCACCATTCATTTTTATCTGGTTGTTCTCAAGTTCGGTTACG
AGATCCATTTGTCTATCTAGTTCAACTTGGAAAATCAACGTATCAGTCG
GGCGGCCTCGCTTATCAACCACCAATTTCATATTGCTGTAAGTGTTTAA
ATCTTTACTTATTGGTTTCAAAACCCATTGGTTAAGCCTTTTAAACTCAT
GGTAGTTATTTTCAAGCATTAACATGAACTTAAATTCATCAAGGCTAATC
TCTATATTTGCCTTGTGAGTTTTCTTTTGTGTTAGTTCTTTTAATAACCA
CTCATAAATCCTCATAGAGTATTTGTTTTCAAAAGACTTAACATGTTCCA
GATTATATTTTATGAATTTTTTTAACTGGAAAAGATAAGGCAATATCTCT
TCACTAAAAACTAATTCTAATTTTTCGCTTGAGAACTTGGCATAGTTTGT
CCACTGGAAAATCTCAAAGCCTTTAACCAAAGGATTCCTGATTTCCACA
GTTCTCGTCATCAGCTCTCTGGTTGCTTTAGCTAATACACCATAAGCAT
TTTCCCTACTGATGTTCATCATCTGAGCGTATTGGTTATAAGTGAACGA
TACCGTCCGTTCTTTCCTTGTAGGGTTTTCAATCGTGGGGTTGAGTAGT
GCCACACAGCATAAAATTAGCTTGGTTTCATGCTCCGTTAAGTCATAGC
GACTAATCGCTAGTTCATTTGCTTTGAAAACAACTAATTCAGACATACA
TCTCAATTGGTCTAGGTGATTTTAATCACTATACCAATTGAGATGGGCT
AGTCAATGATAATTACTAGTCCTTTTCCTTTGAGTTGTGGGTATCTGTAA
ATTCTGCTAGACCTTTGCTGGAAAACTTGTAAATTCTGCTAGACCCTCT
GTAAATTCCGCTAGACCTTTGTGTGTTTTTTTTGTTTATATTCAAGTGGT
TATAATTTATAGAATAAAGAAAGAATAAAAAAAGATAAAAAGAATAGATC
CCAGCCCTGTGTATAACTCACTACTTTAGTCAGTTCCGCAGTATTACAA
AAGGATGTCGCAAACGCTGTTTGCTCCTCTACAAAACAGACCTTAAAA
CCCTAAAGGCTTAAGTAGCACCCTCGCAAGCTCGGGCAAATCGCTGAA
TATTCCTTTTGTCTCCGACCATCAGGCACCTGAGTCGCTGTCTTTTTCG
TGACATTCAGTTCGCTGCGCTCACGGCTCTGGCAGTGAATGGGGGTA
AATGGCACTACAGGCGCCTTTTATGGATTCATGCAAGGAAACTACCCA
TAATACAAGAAAAGCCCGTCACGGGCTTCTCAGGGCGTTTTATGGCGG
GTCTGCTATGTGGTGCTATCTGACTTTTTGCTGTTCAGCAGTTCCTGCC
CTCTGATTTTCCAGTCTGACCACTTCGGATTATCCCGTGACAGGTCATT
CAGACTGGCTAATGCACCCAGTAAGGCAGCGGTATCATCAACAGGCTT
ACCCGTCTTACTGTCGGGAATTGGAAGATCTTCCGGCGAACGTGGCG
AGAAAGGAAGGGAAGAAAGCGAAAGGAGCGGGCGCTAGGGCGCTGG
CAAGTGTAGCGGTCACGCTGCGCGTAACCACCACACCCGCCGCGCTT
AATGCGCCGCTACAGGGCGCGTCCCATTCGCCATTCAGGCTGCGCAA
CTGTTGGGAAGGGCGATCGGTGCGGGCCTCTTCGCTATTACGCCAGC
TGGCGAAAGGGGGATGTGCTGCAAGGCGATTAAGTTGGGTAACGCCA
GGGTTTTCCCAGTCACGACGTTGTAAAACGACGGCCAGTGAGCGCGC
GTAATACGACTCACTATAGGGCGAATTGGAGCTCCACCGCGGTGGCgg
ccgctcgacatttatcccttgcggcgaatacttacagccatgaattcaaaagatctaaagaggagaaag
gatctATGGTGGATAAGAAATACTCAATAGGCttagctatcggcacaaatagcgtcgg
atgggcggtgatcactgatgaatataaggttccgtctaaaaagttcaaggttctgggaaatacagaccgc
cacagtatcaaaaaaaatcttataggggctcttttatttgacagtggagagacagcggaagcgactcgtc
tcaaacggacagctcgtagaaggtatacacgtcggaagaatcgtatttgttatctacaggagatttttcaa

atgagatggcgaaagtagatgatagtttctttcatcgacttgaagagtctttttggtggaagaagacaaga
agcatgaacgtcatcctatttttggaaatatagtagatgaagttgcttatcatgagaaatatccaactatctat
catctgcgaaaaaaattggtagattctactgataaagcggatttgcgcttaatctatttggccttagcgcata
tgattaagtttcgtggtcatttttgattgagggagatttaaatcctgataatagtgatgtggacaaactatttat
ccagttggtacaaacctacaatcaattatttgaagaaaaccctattaacgcaagtggagtagatgctaaa
gcgattctttctgcacgattgagtaaatcaagacgattagaaaatctcattgctcagctccccggtgagaa
gaaaaatggcttatttgggaatctcattgctttgtcattgggtttgacccctaattttaaatcaaattttgatttgg
cagaagatgctaaattacagctttcaaaagatacttacgatgatgatttagataatttattggcgcaaattg
gagatcaatatgctgatttgtttttggcagctaagaatttatcagatgctattttactttcagatatcctaagagt
aaatactgaaataactaaggctcccctatcagcttcaatgattaaacgctacgatgaacatcatcaagac
ttgactcttttaaaagctttagttcgacaacaacttccagaaaagtataaagaaatctttttgatcaatcaaa
aaacggatatgcaggttatattgatgggggagctagccaagaagaattttataaatttatcaaaccaatttt
agaaaaaatggatggtactgaggaattattggtgaaactaaatcgtgaagatttgctgcgcaagcaacg
gacctttgacaacggctctattccccatcaaattcacttgggtgagctgcatgctattttgagaagacaaga
agacttttatccatttttaaaagacaatcgtgagaagattgaaaaaatcttgacttttcgaattccttattatgtt
ggtccattggcgcgtggcaatagtcgttttgcatggatgactcggaagtctgaagaaacaattaccccatg
gaattttgaagaagttgtcgataaaggtgcttcagctcaatcatttattgaacgcatgacaaactttgataaa
aatcttccaaatgaaaaagtactaccaaaacatagtttgctttatgagtattttacggtttataacgaattgac
aaaggtcaaatatgttactgaaggaatgcgaaaaccagcatttctttcaggtgaacagaagaaagccat
tgttgatttactcttcaaaacaaatcgaaaagtaaccgttaagcaattaaaagaagattatttcaaaaaaa
tagaatgttttgatagtgttgaaatttcaggagttgaagatagatttaatgcttcattaggtacctaccatgattt
gctaaaaattattaaagataaagatttttttggataatgaagaaaatgaagatatcttagaggatattgtttta
acattgaccttatttgaagatagggagatgattgaggaaagacttaaaacatatgctcacctctttgatgat
aaggtgatgaaacagcttaaacgtcgccgttatactggttggggacgtttgtctcgaaaattgattaatggt
attagggataagcaatctggcaaaacaatattagatttttttgaaatcagatggttttgccaatcgcaattttat
gcagctgatccatgatgatagtttgacatttaaagaagacattcaaaaagcacaagtgtctggacaagg
cgatagtttacatgaacatattgcaaatttagctggtagccctgctattaaaaaaggtattttacagactgta
aaagttgttgatgaattggtcaaagtaatggggcggcataagccagaaaatatcgttattgaaatggcac
gtgaaaatcagacaactcaaaagggccagaaaaattcgcgagagcgtatgaaacgaatcgaagaa
ggtatcaaagaattaggaagtcagattcttaaagagcatcctgttgaaaatactcaattgcaaaatgaaa
agctctatctctattatctccaaaatggaagagacatgtatgtggaccaagaattagatattaatcgtttaag
tgattatgatgtcgatgccattgttccacaaagtttccttaaagacgattcaatagacaataaggtcttaacg
cgttctgataaaaaatcgtggtaaatcggataacgttccaagtgaagaagtagtcaaaaagatgaaaaa
ctattggagacaacttctaaacgccaagttaatcactcaacgtaagtttgataatttaacgaaagctgaac
gtggaggtttgagtgaacttgataaagctggttttatcaaacgccaattggttgaaactcgccaaatcacta
agcatgtggcacaaattttggatagtcgcatgaatactaaatacgatgaaaatgataaacttattcgaga
ggttaaagtgattaccttaaaatctaaattagtttctgacttccgaaaagatttccaattctataaagtacgtg
agattaacaattaccatcatgcccatgatgcgtatctaaatgccgtcgttggaactgctttgattaagaaat
atccaaaacttgaatcggagtttgtctatggtgattataaagtttatgatgttcgtaaaatgattgctaagtctg
agcaagaaataggcaaagcaaccgcaaaatatttcttttactctaatatcatgaacttcttcaaaacaga
aattacacttgcaaatggagagattcgcaaacgccctctaatcgaaactaatggggaaactggagaaa
ttgtctgggataaagggcgagattttgccacagtgcgcaaagtattgtccatgccccaagtcaatattgtca
agaaaacagaagtacagacaggcggattctccaaggagtcaattttaccaaaaagaaattcggacaa
gcttattgctcgtaaaaaagactgggatccaaaaaaatatggtggttttgatagtccaacggtagcttattc
agtcctagtggttgctaaggtggaaaaagggaaatcgaagaagttaaaatccgttaaagagttactagg
gatcacaattatggaaagaagttcctttgaaaaaaatccgattgactttttagaagctaaaggatataagg
aagttaaaaaagacttaatcattaaactacctaaatatagtctttttgagttagaaaacggtcgtaaacgg
atgctggctagtgccggagaattacaaaaaggaaatgagctggctctgccaagcaaatatgtgaattttt
atatttagctagtcattatgaaaagttgaagggtagtccagaagataacgaacaaaaacaattgtttgtgg
agcagcataagcattatttagatgagattattgagcaaatcagtgaattttctaagcgtgttattttagcagat

	gccaatttagataaagttcttagtgcatataacaaacatagagacaaaccaatacgtgaacaagcaga<br>aaatattattcatttatttacgttgacgaatcttggagctcccgctgcttttaaatattttgatacaacaattgatc<br>gtaaacgatatacgtctacaaaagaagttttagatgccactcttatccatcaatccatcactggtctttatga<br>aacacgcattgatttgagtcagctaggaggtgacggtggctccagatctGCAGGTTCTGGCGA<br>ATTCCAGGCTGTGGTGACTCAAGAACCTAGCGTGACGGTTAGTCCCG<br>GGGGCACGGTCATCCTTACCTGCGGTTCAGGAACAGGAGCGGTTACG<br>TCAGGGCATTACGCAAATTGGTTCCAACAAAAGCCGGGGCAAGCCCC<br>CCGCGCATTGATCTTTGATACCGATAAAAAATATTCTTGGACTCCGGG<br>CCGTTTCTCCGGATCCCTTTTAGGCGCAAAAGCGGCTTTAACCATTAG<br>TGATGCACAACCCGAAGATGAAGCGGAGTATTATTGCTCATTGAGTGA<br>CGTCGATGGCTATCTTTTCGGCGGTGGTACTCAGCTTACCGTTCTGTC<br>AGGCGGGGGGGGAAGCGGCGGAGGTGGATCTGGCGGGGGAGGGTC<br>TGGGGGGGGTGGCAGCCAAGCTGTCGTGACTCAGGAACCCAGCGTG<br>ACCGTGAGCCCCGGCGGAACAGTAATCTTAACATGTGGATCGGGGAC<br>GGGAGCCGTAACGTCCGGGCATTATGCAAACTGGTTCCAGCAAAAGC<br>CAGGTCAGGCACCCCGTGCTCTTATTTTTGACACTGACAAAAAATATTC<br>GTGGACGCCCGGGCGCTTCTCGGGTTCTCTGTTAGGGGCCAAAGCTG<br>CGTTGACCATTAGCGACGCTCAGCCAGAGGACGAAGCGGAATACTAC<br>TGTAGCCTTTCAGACGTAGACGGATACTTATTTGGCGGGGGAACCCAA<br>CTTACCGTTCTTAGCTGATAAcTCGAGGGGGGGCCCGGTACCCAGCTT<br>TTGTTCCCTTTAGTGAGGGTTAATTGCGCGCTTGGCGTAATCATGGTC<br>ATAGCTGTTTCCTGTGTGAAATTGTTATCCGCTCACAATTCCACACAAC<br>ATACGAGCCGGAAGCATAAAGTGTAAAGCCTGGGGTGCCTAATGAGT<br>GAGCTAACTCACATTAATTGCGTTGCGCTCACTGCCCGCTTTCCAGTC<br>GGGAAACCTGTCGTGCCAGGGAAGATCTTCCAGCTT
	pSCW01
	CTCTTCCTTTTTCAAtCTCGAGGATCTCGTGCACAGCttaTTATTGAAGCA<br>TTGCTGAGGGTTATTGTCTCATGAGCGGATACATATTTGAATGTATTTA<br>GAAAAATAAACAAATAGGGGTTCCGCGCACATTTCCCCGAAAAGTGCC<br>ACCTGACGTCATCGAGTCGGATCCGAGTCATTCCTGCAGCGAGTCCAT<br>GGGAGTCAAATAGACAACGATTTGAATTCGCTCTTCCGCTTCCTCGCT<br>CACTGCCTCGCTGCGCTCGGTCGTTCGGCTGCGGCGAGCGGTATCAG<br>CTCACTCAAAGGCGGTAATACGGTTATCCACAGAATCAGGGGATAACG<br>CAGGAAAGAACATGTGAGCAAAAGGCCAGCAAAAGGCCAGGAACCGT<br>AAAAAGGCCGCGTTGCTGGCGTTTTTCCATAGGCTCCGCCCCCCTGAC<br>GAGCATCACAAAAATCGACGCTCAAGTCAGAGGTGGCGAAACCCGAC<br>AGGACTATAAAGATACCAGGCGTTTCCCCCTGGAAGCTCCCTCGTGCG<br>CTCTCCTGTTCCGACCCTGCCGCTTACCGGATACCTGTCCGCCTTTCT<br>CCCTTCGGGAAGCGTGGCGCTTTCTCATAGCTCACGCTGTAGGTATCT<br>CAGTTCGGTGTAGGTCGTTCGCTCCAAGCTGGGCTGTGTGCACGAAC<br>CCCCCGTTCAGCCCGACCGCTGCGCCTTATCCGGTAACTATCGTCTTA<br>AGTCCAACCCGGTAAGACACGACTTATCGCCACTGGCAGCAGCCACT<br>GGTAACAGGATTAGCAGAGCGAGGTATGTAGGCGGTGCTACAGAGTT<br>CTTGAAGTGGTGGCCTAACTACGGCTACACTAGAAGAACAGTATTTGG<br>TATCTGCGCTCTGCTGAAGCCAGTTACCTTCGGAAAAAGAGTTGGTAG<br>CTCTTGGTCCGGCAAACAAACCACCGCTGGTAGCGGTGGTTTTTTTGT<br>TTGCAAGCAGCAGATTACGCGCAGAAAAAAAGCATCTCAAGAAGATTC<br>TTTGATCTTTTCTACGGGGTCTGACGCTCAGTGGAACGAAAACTCACG

TTAAGGGATTTTGGTCATGAGATTATCAAAAAGGATGTTCACCTAGATG
CTTTTAAATTAAAAATGAAGTTTTAAATCAATCTAAAGTATATATGAGTA
AACTTGGTCTGACAGTTACCAATGCTTAATCAGTGAGGCACCTATCTCA
GCGATCTGTCTATTTCGTTCATCCATAGTTGCCTGACTTCCCGTCGTGT
AGATAACTACGATACGGGAGGGCTTACCATCTGGCCCCAGTGCTGCA
ATGATACCGCGAGACCCACGCTCACCGGCTCCAGATTTATCAGCAATA
AACCAGCCAGCCGGAAGGGCCGAGCGCAGAAGTGGTCCTGCAACTTT
ATCCGCCTCCATCCAGTCTATTAATTGTTGCCGGGAAGCTAGAGTAAG
TAGTTCGCCAGTTAATAGTTTGCGCAACGTTGTTGCCATTGCTACAGG
CATCGTGGTGTCACGCTCGTCGTTTGGTAtGGCTTCATTCAGCTCCGGT
TCCCAACGATCAAGGCGAGTTACATGGTCCCCCATGTTGTGCAAAAAA
GCGGTTAGCTCCTTCGGTCCTCCGATCGTTGTCAGAAGTAAGTTGGCC
GCAGTGTTATCACTCATGGTTATGGCAGCACTGCATAATTCTCTTACTG
TCATGCCATCCGTAAGATGCTTTTCTGTGACTGGTGAGTACTCAACCAA
GTCATTCTGAGAATAGTGTATGCGGCGACCGAGTTGCTCTTGCCCGGC
GTCAATACGGGATAATACCGCGCCACATAGCAGAACTTTAAAAGTGCT
CATCATTGGAAAACGTTCTTCGGGGCGAAAACTCTCAAGAATCTTACC
GCTGTTGAGGTCCAGTTCGATGTAACCCACTCGTGCACCCAACTGATC
TTCAGCATCTTTTACTTTCACCAGCGTTTCTGGGTGAGCAAAAACAGGA
AGGCAAAATGCCGCAAAAAAGGGAATAAGGGCGACACGGAAATGTTG
AATACTCATA

Supplementary Table 2: Sequences of DNA oligos used in this study

Substrate strand	Sequence
83_S	5'-CAC ATG CTA TGA GCT GTT GCA ATC TCT CGT ACA ATT AAT AGA CTG GAT GGT GGA TGA CAA AGC TCT ACA CTA GAT ACT CAC AC-3'
83_AS	3-bio-GTG TAC GAT ACT CGA CAA CGT TAG AGA GCA TGT TAA TTA TCT GAC CTA CCA CCT ACT GTT TCG AGA TGT GAT CTA TGA GTG TG-5'
Cap 1	5'-phos-AGT CGC AGC TAT AGG TGG CAT TTC AG-3'
Cap 2	5'-bio-CTG AAA TGC CAC CTA TAG CTG CGA CTC ATG-3'
Arm 1	5-Phos-ACC GAT GTG GTA GGA AGT GAG AAT TGG AGA GTG TGT TTT TTT TTT TTT TTT TTT TTT TTT TTT TTT TTT TTT TTT GAG GAA AGA ATG TTG GTG AGG GTT GGG AAG TGG AAG GAT GGG CTC GAG AGG TTT TTT TTT TTT TTT TTT TTT TTT TTT TTT TTT T-3'
Arm 2	5'-bio-TTT TTT TTT TTT TTT TTT TTT TTT TTT TTT TTT TTT TTT TTT TTT TTT TTT TTT TTT TTT CAC ACT CTC CAA TTC TCA CTT CCT ACC ACA T-3'
$cgRNA_{0.6}$	5'-TTC TAA TAC GAC TCA CTA TAG GAC TCA AGA CGA TAG TTA CGT TTT AGA GCT AGA-3'
$cgRNA_{1.0}$	5'-TTC TAA TAC GAC TCA CTA TAG GTA TCA GCT CAC TCA AAG GGT TTT AGA GCT AGA-3'
$cgRNA_{1.5}$	5'-TTC TAA TAC GAC TCA CTA TAG GCT GCG CAA CTG TTG GGA AGT TTT AGA GCT AGA-3'
$cgRNA_{2.0}$	5'-TTC TAA TAC GAC TCA CTA TAG TAT TAT CCC GTA TTG ACG CGT TTT AGA GCT AGA-3'

Supplementary Table 3: Sequences of guide RNAs used in this study

**Guide RNA**	**Sequence**
cgRNA1	5'-ACA AUU AAU AGA CUG GAU GG-3'
ncgRNA	5'-CAA CAA GUU UGA UUC CAU UG-3'
cgRNA3	5'-CAU UCC UGC AGC GAG UCC AU-3'
cgRNA4	5'-AAA CUC ACG UUA AGG GAU UU-3'
